# Matrix metalloproteinase 15 plays a pivotal role in human first trimester cytotrophoblast invasion and is not altered by maternal obesity

**DOI:** 10.1096/fj.202000773R

**Published:** 2020-07-02

**Authors:** Alejandro Majali‐Martinez, Denise Hoch, Carmen Tam‐Amersdorfer, Jürgen Pollheimer, Andreas Glasner, Nassim Ghaffari‐Tabrizi‐Wizsy, Alexander G. Beristain, Ursula Hiden, Martina Dieber‐Rotheneder, Gernot Desoye

**Affiliations:** ^1^ Department of Obstetrics and Gynecology Medical University of Graz Graz Austria; ^2^ Division of Immunology and Pathophysiology Otto Loewi Research Center Medical University of Graz Graz Austria; ^3^ Department of Obstetrics and Fetal‐Maternal Medicine Medical University of Vienna Vienna Austria; ^4^ Femina Med Center Graz Austria; ^5^ British Columbia Children's Hospital Research Institute University of British Columbia Vancouver BC Canada

**Keywords:** early pregnancy, placenta, trophoblasts, inflammation

## Abstract

Adequate anchoring of the placenta in the uterus through invasion of first trimester cytotrophoblasts (CTB) is required for a successful pregnancy. This process is mediated by matrix metalloproteinases (MMPs) and regulated by the maternal environment. Obesity is known to alter the intrauterine milieu and has been related to impaired invasion. We hypothesized that placental MMP15, a novel membrane‐type MMP, is involved in CTB invasion and regulated by maternal obesity in early pregnancy. Thus, in this study MMP15 was immunolocalized to invasive extravillous and interstitial CTB. MMP15 silencing in chorionic villous explants using two different siRNAs reduced trophoblast outgrowth length (−35%, *P* ≤ .001 and −26%, *P* < .05) and area (−43%, *P* ≤ .001 and −36%, *P* ≤ .01) without altering trophoblast proliferation or apoptosis. Short‐term treatment of primary first trimester trophoblasts with IL‐6 (10 ng/mL), interleukin 10 (IL‐10) (50 ng/mL), and tumor necrosis factor α (TNF‐α) (10 ng/mL) did not affect MMP15 protein levels. Likewise, MMP15 mRNA and protein levels were unaltered between human first trimester placentas from control pregnancies vs those complicated with maternal obesity. Overall, our results suggest that the role of MMP15 in placental development and function in early pregnancy is limited to CTB invasion without being affected by short‐ and long‐term inflammation.

**Abbreviations**
ADAMs: a disintegrin and metalloproteinases
Cleaved K18: caspase‐cleaved cytokeratin 18
CTB: cytotrophoblasts
ECM: extracellular matrix
eCTB: extravillous cytotrophoblasts
GA: gestational age
GW: gestational week
iCTB: interstitial cytotrophoblasts
IL‐6: interleukin 6
IL‐10: interleukin 10
K7: cytokeratin 7
MMPs: matrix metalloproteinases
MMP15: matrix metalloproteinase 15
MT‐MMPs: membrane‐type metalloproteinase
STB: syncytiotrophoblast
TNF‐α: tumor necrosis factor α
vCTB: villous cytotrophoblasts

## 1 INTRODUCTION

Successful pregnancy requires adequate placental development and function. Already in the first trimester, placenta‐specific cytotrophoblasts (CTB) differentiate along several pathways to fulfill a wide range of distinct functions that enable placental, and hence, fetal development.^1^ Proliferative villous cytotrophoblasts (vCTB) can fuse to form the syncytiotrophoblast (STB), a multinucleated layer representing the classical placental barrier and involved in nutrient transport to the fetus as well as hormone synthesis and secretion.^2^ At contact sites between placental villi and the uterine basement membrane, vCTB further proliferate and form cell columns. At the distal part of these columns vCTB subsequently differentiate into invasive extravillous cytotrophoblasts (eCTB).^3^ Decidua invading eCTB, that is, interstitial cytotrophoblasts (iCTB), also reach and remodel the spiral arteries, a process required for the establishment of sufficient blood supply to the fetus.^4^

eCTB invasive potential is determined by their capacity to degrade the surrounding extracellular matrix (ECM). Therefore, eCTB express a broad repertoire of proteases, including serine‐proteases, cathepsins, a disintegrin and metalloproteinases (ADAMs), and matrix metalloproteinases (MMPs).^5^, ^6^ The latter are a family of 23 endopeptidases whose substrates include the majority of ECM components.^7^ MMPs are synthesized as zymogens and can either be secreted or anchored to the plasma membrane, that is, membrane‐type (MT)‐MMPs.^8^ Membrane‐type metalloproteinase (MT‐MMPs) can be specifically expressed at the forefront of invading cells, and thus, enable site directed degradation of ECM during invasion.^9^

We have previously highlighted the importance of MT‐MMPs in the pathophysiology of pregnancy.^10^ Among them, only MMP14 has been extensively characterized in human first trimester placenta, where it plays a role in trophoblast proliferation, migration, and invasion.^11^, ^12^ Besides MMP14, MMP15 is the only MT‐MMP expressed in the placenta during early pregnancy.^10^, ^13^, ^14^ However, the specific role of MMP15 in first trimester placenta remains poorly understood.

CTB invasion is strictly regulated in a spatiotemporal manner.^15^ This regulation is mediated by autocrine placenta‐derived signals such as human chorionic gonadotropin^16^ and placental growth hormone.^17^ The intrauterine environment also plays a crucial role. Several paracrine factors, including endothelin‐1,^18^ tumor necrosis factor (TNF)‐α,^19^ and transforming growth factor (TGF)‐β^20^ modulate CTB invasion. Interestingly, these stimuli also regulate MMPs.^11^, ^21^ Hence, derangements in the intrauterine environment affecting MMPs might in turn affect CTB invasion and, ultimately, alter placental development.^22^

Metabolic and endocrine changes in obesity induce a chronic low grade inflammatory environment characterized by increased levels of C‐reactive protein, TNF‐α and interleukin (IL)‐6 as well as decreased levels of interleukin 10 (IL‐10).^23^, ^24^ Similarly, obesity in pregnancy is associated with low‐grade inflammation and oxidative stress.^25^ Obesity is known to alter placenta development and function, entailing adverse consequences for both fetal and maternal health.^26^ Obese women have an increased risk for early pregnancy loss and preeclampsia, and both disorders originate in early pregnancy due to impaired CTB invasion and MMP dysregulation.^27^ However, the molecular mechanisms explaining how obesity might alter CTB invasion in the first trimester of pregnancy have been incompletely elucidated.

Here, we describe MMP15 placental location in early human pregnancy and assess whether MMP15 plays a pivotal role in CTB invasion. We also hypothesize that maternal obesity affects MMP15 regulation. Therefore, we tested whether placental MMP15 expression and protein levels are altered by in vitro short‐term exposure to obesity‐associated pro‐ and anti‐inflammatory cytokines (IL‐6, IL‐10, and TNF‐α), as well as by long‐term exposure to the intrauterine environment characteristic of obesity in vivo.

## 2 MATERIAL AND METHODS

### 2.1 Ethics statement

The study was approved by the institutional review board and ethical committee of the Medical University of Graz (24‐129 ex 11/12), the Medical University of Vienna (084/2009) and the University of British Columbia (H13‐00640).

### 2.2 Tissue collection

Women participating in the study were recruited upon signing an informed consent. Gestational age (GA) was determined by ultrasound measurement of the crown rump length. Women's height and weight were measured at the time of recruitment and used to calculate maternal body mass index (BMI). Women under medication or with comorbidities were excluded from the study. Human first trimester placental tissue (gestational week (GW) 5‐11, n = 59) from lean (BMI < 25 kg/m^2^) and obese (BMI ≥ 30 kg/m^2^) women and *decidua basalis* (GW 8‐10, n = 4) were collected after elective pregnancy termination for psychosocial reasons.

### 2.3 Placental chorionic villous explants

Human first trimester chorionic villous explants were established as previously described.^28^ Briefly, placental villi from the periphery were dissected under the microscope, washed in cold phosphate‐buffered saline (PBS), and placed on cell culture inserts (EMD Millipore, Darmstadt, DE) pre‐coated with 200 µL of growth‐factor reduced Matrigel (Corning, Bedford, MA, USA). Thereafter, 400 µL of Dulbecco's Modified Eagle's (DMEM)/Ham's F12 medium (Gibco, Invitrogen, Carlsbad, CA, USA) supplemented with penicillin/streptomycin (Gibco) were added to the outer chamber. Explants were maintained overnight in a humidified incubator at 37°C, 5% CO_2_ and 3% O_2_ to allow anchoring. To knockdown MMP15 expression, explants were incubated for 24 hours with 200 µL of DMEM/Ham's F12 medium containing non‐targeting (NT)‐siRNA (control, 100nM, Qiagen, Hilden, DE) or two different MMP15‐specific siRNAs (si5‐ and si6‐siRNA, 100nM, Qiagen). Knockdown efficiency was validated by RT‐qPCR (see RNA isolation and RT‐qPCR analysis). Subsequently, trophoblast outgrowth was monitored for 72 hours using a microscope (Nikon SMZ 745, Vienna, AU) coupled to a camera (Moticam 580, Hong Kong, CH). Outgrowth length, measured as the distance between the villous margin and the front of the migrating cell sheet, and outgrowth area were calculated using ImageJ software.

### 2.4 Trophoblast isolation and culture

Human first trimester trophoblast were isolated as described elsewhere.^29^ Briefly, placental villi were digested with Dispase/DNAse and Trypsin (Gibco, Invitrogen, Carlsbad, CA, USA). After Percoll (Gibco) gradient centrifugation, cells were incubated with magnetic beads conjugated with anti‐CD90 and anti‐CD45 (Dako, Glostrup, DK) to remove fibroblasts and common leukocyte‐antigen expressing cells. Purity of trophoblast isolations was determined by cytokeratin 7 (K7) (Dako, 1:750) and HLA‐G (BD‐Biosciences, Bedford, MA, USA, 1:500) immunostaining. Only preparations with a purity ≥ 95% were used.

Immediately after isolation, trophoblasts were seeded in gelatin pre‐coated plates and maintained for 48 hours in Keratinocyte medium (KCM, Gibco) with penicillin/streptomycin (Gibco), KCM supplements (Gibco), and 10% (v/v) fetal calf serum (FCS, Thermo Scientific, Scientific, Rockford, IL, USA) in a humidified incubator at 37°C, 5% CO_2_. Subsequently, cells were cultured under low serum conditions (2% (v/v) FCS) for 24 hours and further incubated in the absence (control) or presence of IL‐6 (10 ng/mL, Sigma Aldrich, St. Louis, MO, USA), IL‐10 (50 ng/mL, Sigma Aldrich), or TNF‐α (10 ng/mL. Sigma Aldrich). Cytokine incubation was performed for 24 hours as previously demonstrated to be sufficient for regulation of MMPs.^30^

### 2.5 RNA isolation and RT‐qPCR analysis

Total RNA from first trimester placental tissue and chorionic villous explants was isolated using the RNeasy mini kit (Qiagen, Hilden, DE). RNA (250 ng) was reverse transcribed to cDNA with SuperScript II Reverse Transcriptase (Life Technologies, Carlsbad, CA, USA) as per manufacturer's guidelines. *MMP15* and *HLA‐G* expression was determined by RT‐qPCR using FAM‐labeled TaqMan gene expression assays (Life Technologies, *MMP15*: Hs00233997_m1; *HLA‐G*: Hs00365950_g1), TaqMan universal PCR master mix (Life Technologies) and the CFX96 and CFX384 real‐time PCR detection systems (BioRad Laboratories, Hercules, CA, USA). To test for compensatory changes of MMP15 knockdown by another key MT‐MMP, *MMP14*, and *β‐actin* expression were also quantified (Life Technologies, *MMP14*: Hs01037003_g1; *ACTB*: Hs01060665_g1). Ct values were automatically generated by the BioRad CFX Manager 3.0 software and relative gene expression was expressed as −ΔCt or calculated by the 2^−ΔΔCt^ method, with *HLA‐G* as the reference gene. Fetal sex was determined based on *XIST* (Life Technologies, Hs01079824_m1) and *DDX3Y* (Life Technologies, Hs00965254_gH) expression as described elsewhere.^31^

### 2.6 Protein quantification by western blotting

Placental and trophoblast protein lysates were prepared in RIPA buffer (Sigma Aldrich) containing protease inhibitors (Roche, Mannheim, DE). Protein concentration was determined by bicinchoninic acid assay (Thermo Scientific). Western blotting was performed as described elsewhere.^18^ The specific primary and secondary antibodies used in this study are shown in Supporting Table S1. Results were normalized to HLA‐G protein levels. A placental protein lysate was used as internal calibrator to account for inter‐blot variation.

### 2.7 Immunofluorescence

First trimester placental and decidual tissues as well as Matrigel chorionic villous explants were fixed in 2%‐4% (v/v) paraformaldehyde (PFA), paraffin‐embedded and sectioned at 5 µm onto glass slides. Subsequently, slides were deparaffinized in xylene (Thermo Scientific) and rehydrated with decreasing concentrations of ethanol. For antigen retrieval, tissue sections were submerged in Reveal Decloaker buffered solution (Biocare Medical, Pacheco, CA, USA) and heated in a microwave for 5 x 2‐min intervals. Sections were rinsed in PBS and blocked with 5% of normal goat serum (Thermo Scientific) for 1 hour prior to addition of the appropriate combination of primary and secondary antibodies (Supporting Table S1). Slides were mounted with Prolong gold anti‐fade reagent with 4′,6‐diamidino‐2‐phenylindole (DAPI, Life Technologies) to counterstain the nuclei. Images were acquired and analyzed using a Zeiss Axio Z1 microscope equipped with an Axiocam (Zeiss, Jena, DE) and with the ZEN Software (Zeiss), respectively.

### 2.8 Statistics

Statistical analysis was performed using GraphPad 5.1 and IBM SPSS Statistics 25. After testing for normal distribution (Kolmogórov‐Smirnov), parametric and nonparametric data were analyzed by *t* test or Mann‐Whitney U test, respectively, or by Fisher's exact test as appropriate. Associations between MMP15 levels, BMI and GA were determined by Pearson's and Spearman's correlation coefficients, or by using a multivariate linear regression model adjusted for smoking status and fetal sex. This model included BMI and GA, both as continuous variables, as the exposures, and MMP15 mRNA, as well as MMP15 and HLA‐G protein levels as the outcomes. *P* < .05 was considered statistically significant.

## 3 RESULTS

### 3.1 MMP15 localizes to eCTB and iCTB in early human pregnancy

**FIGURE 1:**
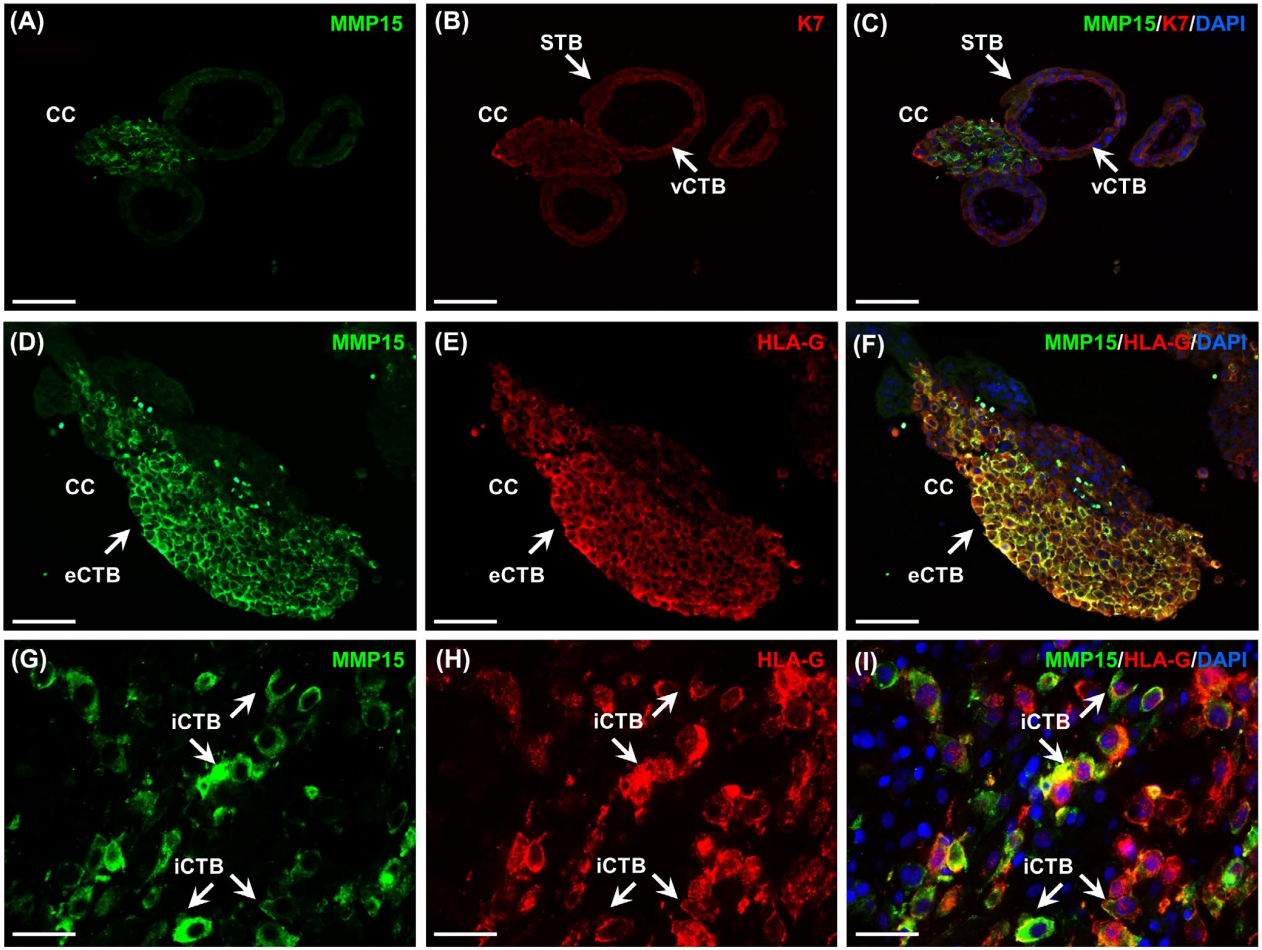
Extravillous (eCTB) and interstitial cytotrophoblasts (iCTB) are the main source of MMP15 in human early pregnancy. MMP15 immunofluorescence staining was performed in serial sections of human placental tissue (GW 6‐10) and *decidua basalis* (GW 7‐10). MMP15 localized to eCTB in the cell columns (CC, A and D) as well as to iCTB invading through the decidua (G). Villous cytotrophoblasts (vCTB) and syncytiotrophoblast (STB) were identified by cytokeratin 7 (K7) immunostaining (B and C). eCTB and iCTB were further identified by positive immunostaining for HLA‐G (E and H). Image overlay showed placental and decidual MMP15 and HLA‐G co‐localization (F and I). Nuclei were counterstained with DAPI. Scale bar: 100 µm (A‐F) and 50 µm (G‐I)

In order to identify a potential role of MMP15 in early placental development, we localized MMP15 by immunofluorescence staining in human first trimester placenta (Figure 1A,D). Sections were double‐stained for K7, a marker for all trophoblast populations (K7, Figure 1B and C), or HLA‐G, a specific eCTB marker (Figure 1E). MMP15 was predominantly located in the distal part and the front of the cell columns and co‐localized with HLA‐G, suggesting that eCTB might be the main source of placental MMP15 in early pregnancy (GW 6‐10, n = 4, Figure 1F). This notion is supported by the positive correlation (*r* = .79; *P* < .0001) between placenta *MMP15* and *HLA‐G* expression (Supporting Figure S1, GW 7‐11, n = 42). Therefore, HLA‐G was further used for data normalization.

In first trimester decidua samples (GW 7‐10, n = 4) iCTB, also identified by HLA‐G positive staining, were the only cell population expressing MMP15 (Figure 1G‐I).

### 3.2 MMP15 is involved in CTB invasion, but not in proliferation or apoptosis

**FIGURE 2:**
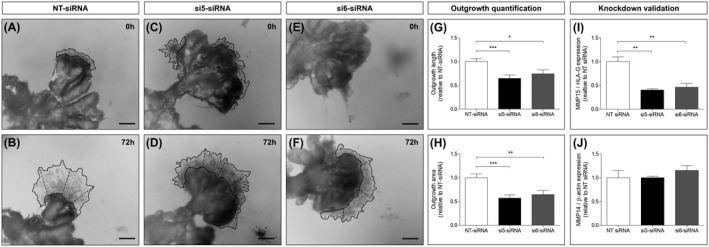
MMP15 silencing decreases trophoblast outgrowth in first trimester chorionic villous explants. Dissected chorionic villi from human first trimester placentas (GW 6‐10) were incubated with non‐targeting (NT)‐siRNA (control, A) or with two different MMP15‐specific siRNA (si5‐ and si6‐siRNA, C and E) for 24 h. Trophoblast outgrowth was monitored for 72 h, and quantified as the length between the villous tip and the front of the outgrowing sheet (dotted lines, B, D, and F), or as the outgrowth area (solid line, B, D, and F). Results were calculated relative to NT‐siRNA (G and H), arbitrarily set to 1, and expressed as mean ± SEM. Knockdown efficiency was validated by MMP15 (I) and MMP14 (J) RT‐qPCR, with HLA‐G and β‐actin as housekeeping genes, respectively. n = 4 different placentas, 3‐11 villi for each condition. *P < .05; **P ≤ .01; ***P ≤ .001 vs NT‐siRNA

To determine a potential role of MMP15 in CTB invasion, MMP15 was silenced in human first trimester placental chorionic villous explants (GW 8‐10, n = 4, Figure 2A‐F) using two different siRNAs (si5‐siRNA and si6‐siRNA). CTB invasion was analyzed by quantifying outgrowth length, that is, the distance between the villous margin and the outer edge of the cell column (Figure 2B,D,F, dotted lines), as well as the outgrowth area (Figure 2B,D,F, solid line) after 72 hours.

When compared to the control, that is, non‐targeting (NT)‐siRNA, both si5‐ and si6‐siRNA significantly decreased trophoblast outgrowth length (−35%; *P* ≤ .001 and −26%; *P* < .05; Figure 2G) and outgrowth area (−43%; *P* ≤ .001 and −36%; *P* ≤ .01; Figure 2H). MMP15 knockdown efficiency was confirmed by RT‐qPCR, with both siRNAs showing a downregulation of MMP15 expression (−60% and −54%; *P* ≤ .01; vs NT‐siRNA, Figure 2I) without affecting MMP14 mRNA levels (Figure 2J).

**FIGURE 3:**
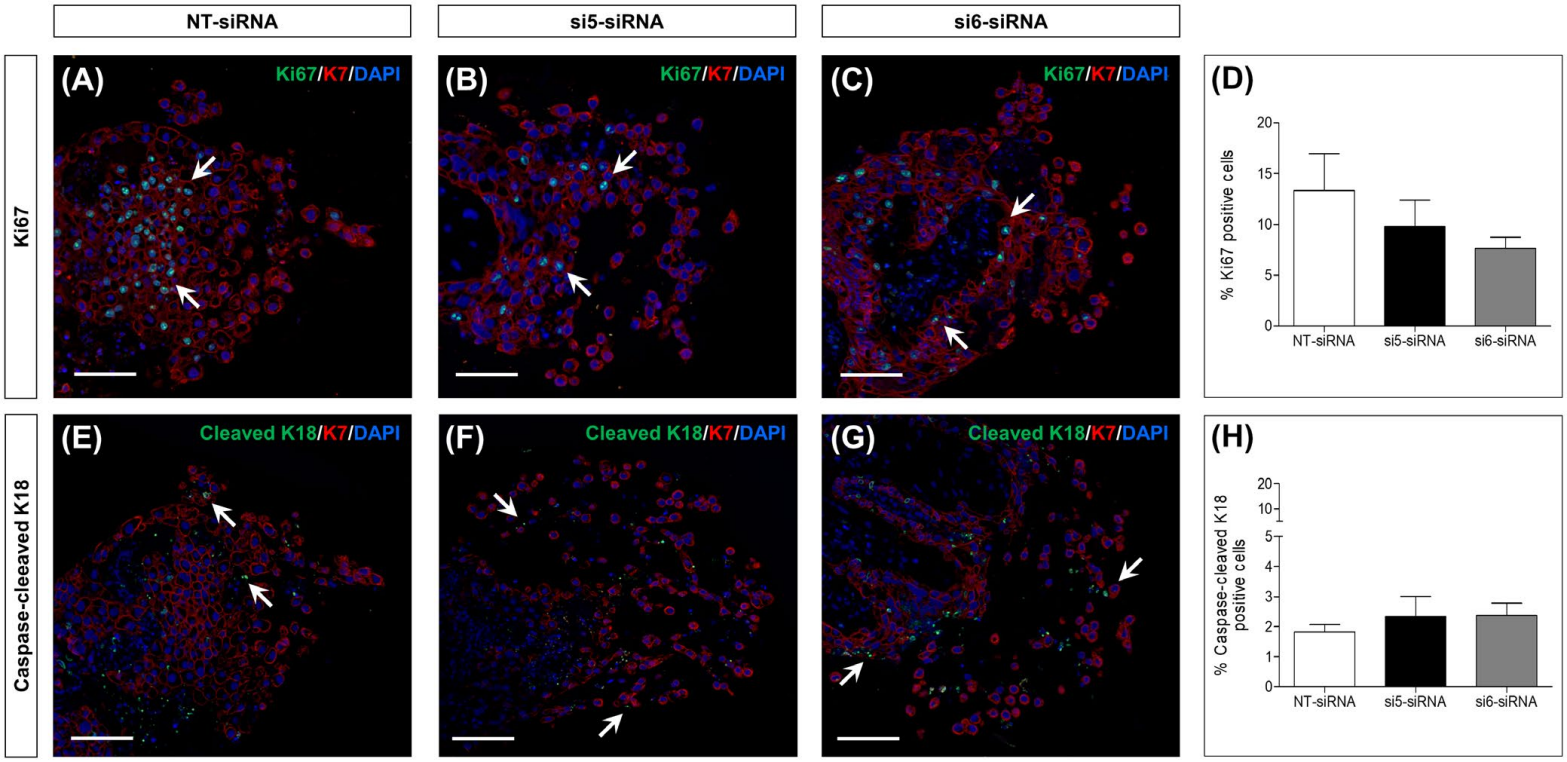
MMP15 silencing does not affect trophoblast proliferation and apoptosis in first trimester chorionic villous explants. Dissected chorionic villi from human first trimester placentas (GW 6‐10) were incubated for 24 h with non‐targeting (NT)‐siRNA (control) or with two different MMP15‐specific siRNA (si5‐ and si6‐siRNA). After 72 h, trophoblast proliferation and apoptosis were determined by Ki67 (A‐C) and caspase‐cleaved cytokeratin 18 (cleaved K18, E‐G) immunofluorescence staining, respectively. Results were calculated as percentage of Ki67 and cleaved K18 positive cells (arrows) relative to the number of cytokeratin 7 (K7) positive cells (D and H). Data are expressed as mean ± SEM. n = 4 different placentas, 2‐3 cell columns for each condition

Trophoblast outgrowth might also reflect CTB proliferation and apoptosis. Therefore, we additionally investigated a potential role of MMP15 in these biological processes. For that purpose, first trimester placental explants were immunostained for Ki67 (Figure 3A‐C) and caspase‐cleaved cytokeratin 18 (cleaved K18, Figure 3E‐G) under silencing of MMP15. Proliferation and apoptosis were calculated as the percentage of Ki67 (Figure 3D) or cleaved K18 positive cells (Figure 3H) relative to number of K7 positive cells, respectively. MMP15 knockdown had no effect on CTB proliferation or apoptosis.

### 3.3 MMP15 is not affected by short‐term exposure to inflammatory mediators

**FIGURE 4:**
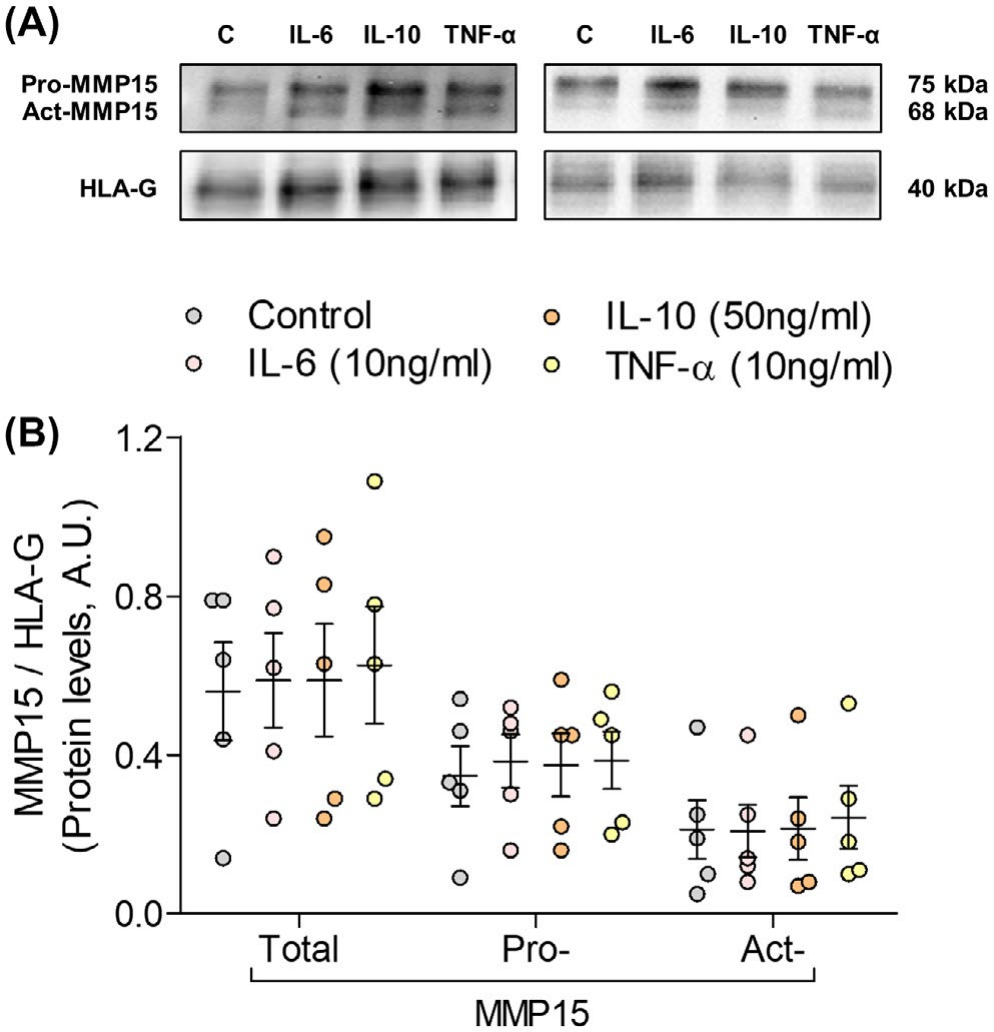
MMP15 is unaffected by short‐term exposure to obesity‐associated pro‐inflammatory cytokines. Primary first trimester trophoblasts (GW 7‐9) were incubated in the absence (control) or the presence of IL‐6 (10 ng/mL), IL‐10 (50 ng/mL), or TNF‐α (10 ng/mL). MMP15 protein levels (pro‐MMP15, active (act)‐MMP15, and total‐MMP15: pro + act‐MMP15) were determined by Western blotting (A). Results were normalized to HLA‐G protein levels and expressed as mean ± SEM (B). n = 5 different placentas, each treatment was assayed in duplicates

To assess whether components of the pro‐inflammatory environment associated with obesity modulate MMP15 protein levels, primary human first trimester trophoblast (GW 7‐9, n = 5) were incubated with IL‐6, IL‐10, and TNF‐α for 24 hours. Pro‐ and active (act)‐MMP15 as well a total‐MMP15 (sum of pro‐ and act‐MMP15) were determined by Western blotting (Figure 4A). There was no difference in total‐, pro‐, or act‐MMP15 levels between control and the cytokine treatments (Figure 4B). MMP15 ratios (act‐ to pro‐, pro‐ to total‐, and act‐ to total‐MMP15), which reflect changes in MMP15 activation, were also not altered by the cytokines (Supporting Figure S2A).

### 3.4 MMP15 is not affected by maternal obesity

In order to test whether long‐term exposure to the obesity‐associated intrauterine environment might affect MMP15, we quantified MMP15 mRNA and protein levels in first trimester placental tissue from lean (BMI < 25, GW 5‐11, n = 24) vs obese women (BMI ≥ 30, GW 5‐10, n = 18). Maternal and fetal characteristics are shown in Table 1.

**Table 1:** Characteristics of the study cohort used to determine the effect of maternal obesity on MMP15 regulation

Characteristics	Lean	Obese	*P* value
Sample size (n)	24	18	
Body mass index (BMI)	20.1 ± 1.5	34.2 ± 3.7	**<.001**
Gestational age (days)	60.7 ± 13.7	55.8 ± 11.7	.232
Maternal age (years)	28.7 ± 8.1	29.0 ± 6.6	.901
Fetal sex (male/female)	10/14	12/6	.131
Smoker (yes/no)	2/22	8/10	**.010**

Note

Data presented as mean ± SD. Statistical analysis included Mann‐Whitney, *t* test and Fisher's exact test. Statistically significant results are marked in bold.

**FIGURE 5:**
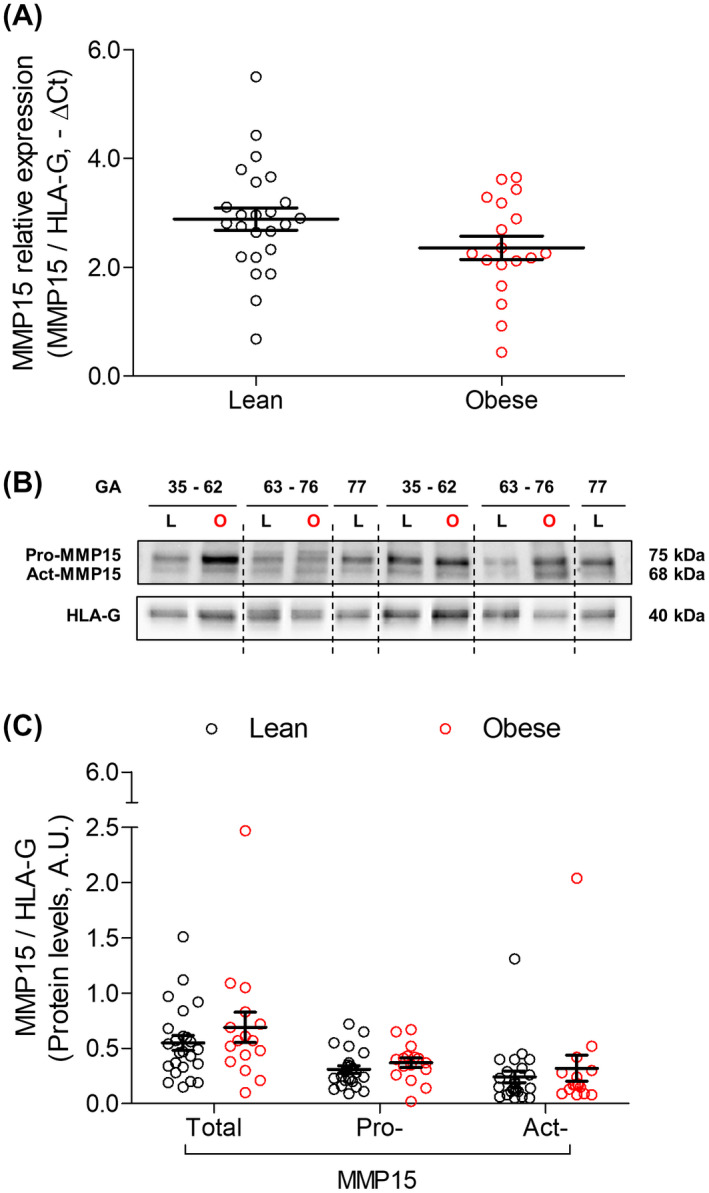
MMP15 is unaffected by long‐term exposure to obesity‐associated intrauterine environment. MMP15 expression and protein levels (pro‐MMP15, active (act)‐MMP15, and total‐MMP15: pro + act‐MMP15) were determined in first trimester placental tissue from lean (GW 5‐11, n = 24) and obese (GW 5‐10, n = 18) women by RT‐qPCR (A) and Western blotting (B), respectively. Results were normalized to HLA‐G expression (−ΔCt) and protein levels and expressed as mean ± SEM. GA, gestational age (days)

Maternal obesity did not affect placental MMP15 mRNA expression (Figure 5A) nor total‐, pro‐, and act‐MMP15 protein levels (Figure 5B,C), or their ratios (Supporting Figure S2B). Data stratification also showed no correlation between MMP15 mRNA and protein levels and maternal BMI (Supporting Figure S3). However, GA negatively correlated with total‐ (*r* = −.454; *P* = .003), pro‐ (*r* = −.339; *P* = .032), and act‐MMP15 (*r* = −.442; *P* = .004) protein levels (Supporting Figure S4). These results were confirmed using a multivariate lineal regression model adjusting for smoking status and fetal sex with maternal BMI and GA as the exposures. In this model, only GA predicted total‐ (*β* = −.356; *P* = .031) and pro‐MMP15 (*β* = −.320; *P* = .050) protein levels. This downregulation was not accounted for by a decreased proportion of eCTB, since HLA‐G protein levels were not affected by GA (Table 2). There was no statistically significant (*P* > .10) interaction between GA and maternal BMI.

**Table 2:** Associations between MMP15 and HLA‐G levels and maternal BMI or gestational age in human first trimester placenta

Outcomes	Sample size (n)	Maternal BMI		Gestational age (days)	
		Standardized coefficient (*β*)	*P* value	Standardized coefficient (*β*)	*P* value
MMP15 expression	42	−0.113	.527	0.053	.744
Total MMP15 protein levels	40	0.180	.287	**−0.335**	**.033**
Pro‐MMP15 protein levels	40	0.244	.154	**−0.301**	**.054**
Act‐MMP15 protein levels	40	0.102	.573	−0.257	.120
HLA‐G protein levels	40	−0.076	.674	0.165	.317

Note

Multivariate linear regression model adjusted for smoking status and fetal sex. Statistically significant results are marked in bold.

Abbreviation: BMI, body mass index.

## 4 DISCUSSION

MMPs are required for invasion processes such as CTB invasion.^32^ However, studies addressing MMPs in human first trimester placenta have traditionally focused on secreted MMPs, that is, MMP2 and MMP9.^33^, ^34^ MT‐MMPs have recently gained attention in early pregnancy, when MMP14 has been extensively characterized.^11^, ^12^ However, the role of MMP15, the other MT‐MMP highly expressed in human first trimester placenta,^10^ has remained elusive.

We revealed that in first trimester placental tissue MMP15 is exclusively present on the cell columns which anchor the placenta to the uterus. Cell columns are comprised by two different CTB populations, proliferative vCTB, located in immediate proximity of the villus, and invasive eCTB found on the distal part of the column.^35^ Co‐localization of MMP15 with HLA‐G, a specific eCTB marker,^36^ confirmed that MMP15 location is restricted to eCTB. This contrasts with MMP14, which in first trimester placenta is found not only in eCTB, but also in vCTB, STB and the endothelial cells of fetal capillaries.^12^

Previously, MMP15 expression was described in CTB isolated from human decidual tissue.^37^ Here, we showed that iCTB are the main source of MMP15 in first trimester *decidua basalis*. MMP15‐specific substrates include several decidua ECM proteins such as collagen I and IV, laminin, and vitronectin.^38^, ^39^

To identify its functional implication in early pregnancy, we silenced MMP15 expression in human chorionic villous explants. This led to a specific decrease in MMP15 expression without upregulating the other MT‐MMP present in first trimester placenta, that is MMP14, as a compensatory mechanism. Reduction of MMP15 restricted trophoblast outgrowth without affecting proliferation and apoptosis. These results argue for an involvement of MMP15 in CTB invasion. However, we cannot distinguish between a direct MMP15 effect and an indirect effect through activation of pro‐MMP2.^40^ Likewise, the activity of other ECM‐degrading enzymes, for example, ADAMs, cathepsins and serine proteases,^5^, ^6^ could also explain why trophoblast outgrowth was not completely abrogated upon MMP15 silencing. Interestingly, MMP15 inhibits apoptosis in several tumor cell lines.^41^ Absence of this apoptosis inhibiting effect in our study may reflect the difference between the physiological cell cycle regulation of CTB vs the excessive proliferation of tumor cells.

Maternal obesity is a pregnancy condition characterized by an intrauterine milieu altered already in early pregnancy.^25^ Recent studies have shown that maternal obesity alters uterine natural killer cell function, which hinders trophoblast outgrowth in human chorionic villous explants and delays remodeling of the spiral arteries in mice.^42^, ^43^, ^44^ Therefore, in the present study, we tested whether the obesity associated pro‐inflammatory environment alters MMP15. For that purpose, we focused on IL‐6, IL‐10, and TNF‐α, key pro‐ and anti‐inflammatory cytokines known to be dysregulated in obesity.^45^ Treatment of first trimester trophoblast with these cytokines did not alter MMP15 protein levels or MMP15 activation. However, these in vitro results have some limitations. Cytokine treatments were performed under 21% O_2_, which is above the physiological oxygen tension in early pregnancy (2.5%‐6.5% O_2_).^46^ Moreover, although the cytokine concentrations used are within the pathophysiological range,^47^, ^48^, ^49^ dose and time‐dependent effects as well as an interplay between them cannot be ruled out.

In order to study potential obesity effects on MMP15 in a more physiological context we considered maternal obesity as a chronic condition and also tested the effect of long‐term exposure to the obesity‐associated intrauterine environment on MMP15 in vivo levels. However, we did not find differences on placental MMP15 expression, protein levels, or activation between the lean and the obese cohort. Thus, both in vitro as well as in vivo results strongly suggest lack of an obesity effect on placental MMP15 in first trimester human pregnancy. However, we cannot rule out that obesity affects any other of the complex mechanisms governing CTB invasion, independent on MMP15. We recently demonstrated an influence of maternal obesity on placental cell cycle in early pregnancy.^50^ Thus, other biological processes in the first trimester might be affected by maternal obesity and indirectly have an impact on CTB invasion.

In the present study, we defined maternal obesity based on BMI. Other obesity‐associated factors such as leptin might have been better proxy measures of maternal obesity, since leptin can inhibit inflammation‐mediated MMP2 and MMP9 activation at least at term of pregnancy.^51^

The major strength of this study is the use of first trimester placental and decidual tissue as well as villous chorionic explants, where the complex interactions and communication between the different cell types comprising the human placenta in early pregnancy is preserved. Moreover, GA was considered as a continuous variable, which allows studying potential changes in effects within the first trimester of pregnancy. Primary human trophoblasts are a better model than first trimester trophoblast cell lines and were used here. The main limitation is the lack of in depth characterization of the pro‐inflammatory and metabolic parameters within the obese cohort, but this was outside the scope of the study. Other decidual CTB populations that might be a source of MMP15, that is, iCTB reaching the spiral arteries and decidual glands, were also not studied.

To the best of our knowledge, this is the first study assessing placental MMP15 location and function in human early pregnancy. Altogether, our results suggest that MMP15 is highly restricted to the invasive compartment in human first trimester placenta. Despite the broad repertoire of proteases found in trophoblasts, MMP15 appears to be crucial for CTB invasion. Maternal obesity does not affect MMP15 levels in early pregnancy. Nevertheless, we observed a downregulation of MMP15 protein levels with increasing GA. This reduction is not the result of a decreased proportion of eCTB, since HLA‐G protein levels did not change with GA. Thus, our results suggest that MMP15 regulation is fine‐tuned both spatially and temporally in the first trimester of pregnancy.

## CONFLICT OF INTEREST

The authors have no conflict of interest to declare.

## AUTHOR CONTRIBUTIONS

A. Majali‐Martinez participated in the conception and design of the study, performed the majority of the experiments and data analysis, and wrote the first draft of the manuscript. D. Hoch was involved in tissue collection, processing, and characterization. C. Tam‐Amersdorfer performed the immunostainings in first trimester placental tissue. J. Pollheimer performed the immunostainings in first trimester decidua and critically reviewed the manuscript. A. Glasner provided the first trimester material. N. Ghaffari‐Tabrizi‐Wizsy critically reviewed the manuscript. AG Beristain planned the experiments with chorionic villous explants and critically reviewed the manuscript. U. Hiden, M. Dieber‐Rotheneder, and G. Desoye participated in the conception and design of the study, were involved in data discussion and critically reviewed the manuscript.

## Supporting information

Fig S1‐S4

Table S1
