# Supplementary material for: Matrix metalloproteinase 15 plays a pivotal role in human first trimester cytotrophoblast invasion and is not altered by maternal obesity
Source: FASEB J. 2020 Jul 2;34(8):10720–30. doi: 10.1096/fj.202000773R (PMC7496590; doi:10.1096/fj.202000773R)
Supplement: Supplementary file 1 — Fig S1‐S4 [file FSB2-34-10720-s001.docx]

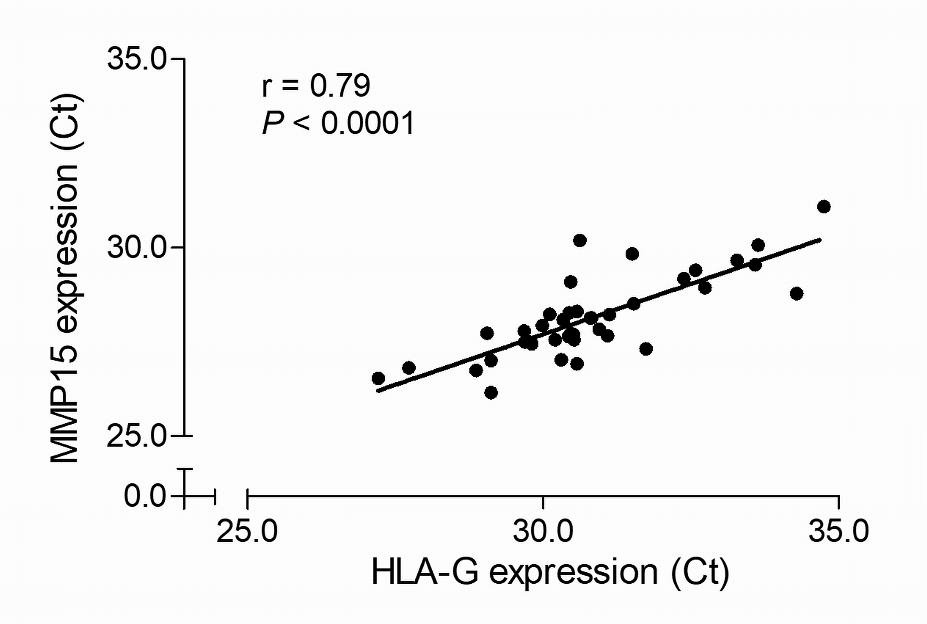


**Supplementary Figure S1**. **Positive correlation between placental MMP15 and HLA-G expression.** MMP15 and HLA-G mRNA levels were determined in first trimester placental tissue (GW 5-11, n = 42) by RT-qPCR. Results are shown as Ct values. Pearson’s correlation was used to determine associations between MMP15 and HLA-G.


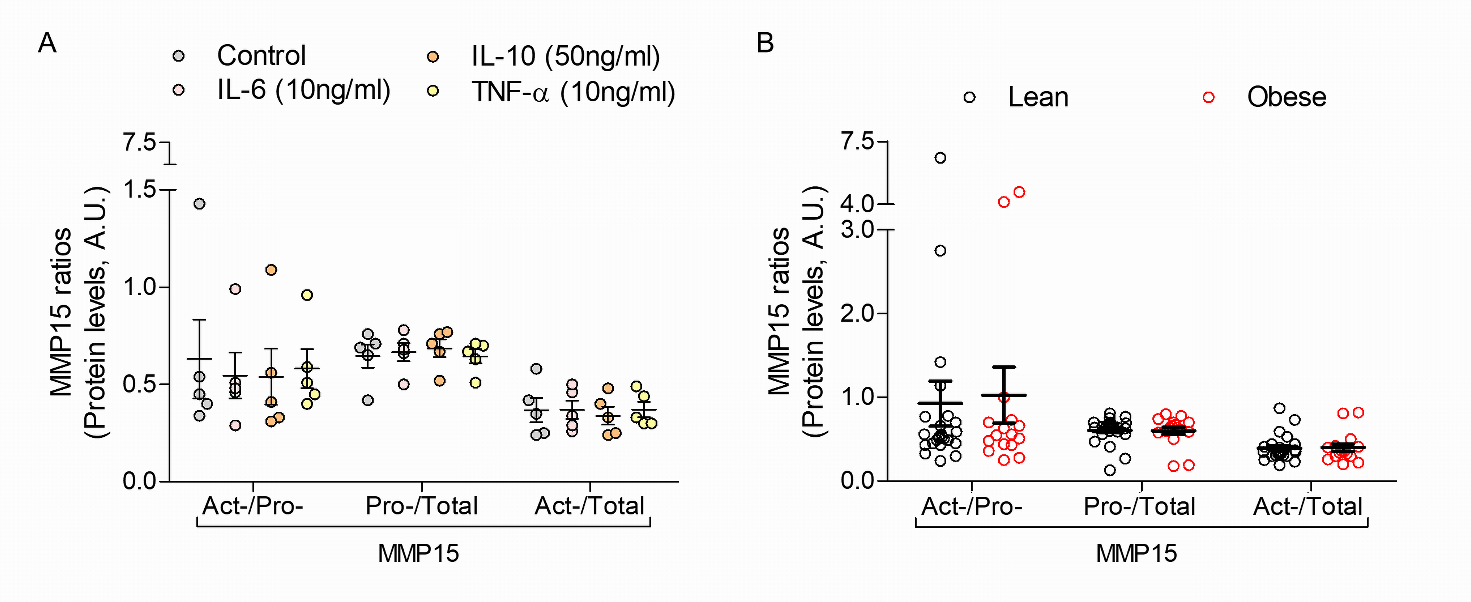


**Supplementary Figure S2. MMP15** **activation is not affected by short-term exposure to obesity-associated pro-inflammatory cytokines or by long-term exposure to maternal obesity.** Act- to pro-, pro- to total and act- to total MMP15 protein ratios were calculated as a proxy measure for MMP15 activation in primary first trimester trophoblasts (GW 7-9) incubated in the absence (control) or the presence of IL-6 (10ng/ml), IL-10 (50ng/ml) or TNF-α (10ng/ml) (**A**). These ratios were also determined in first trimester placental tissue from lean (GW 5-11, n = 24) and obese (GW 5-10, n = 18) women (**B**).


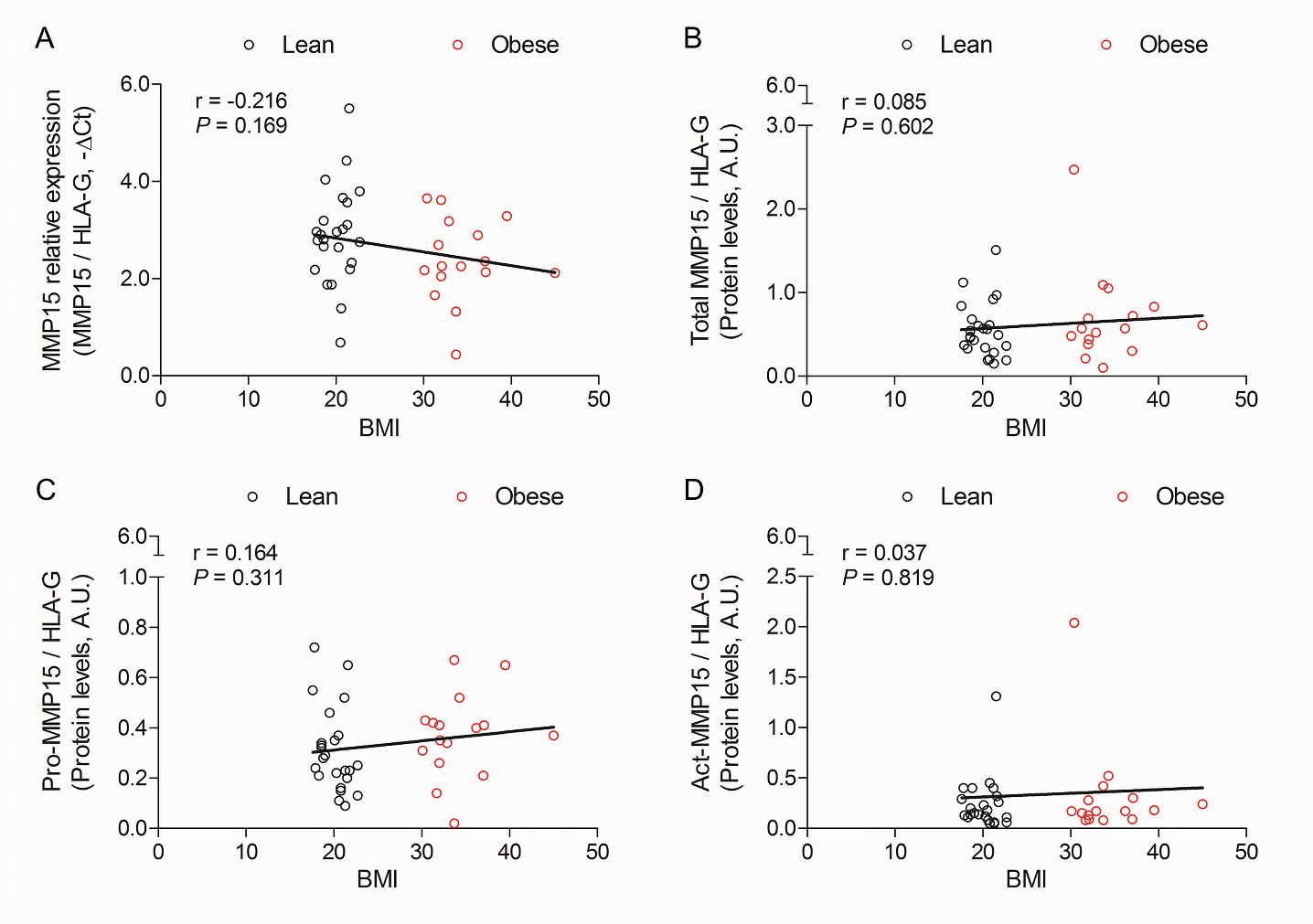
**Supplementary Figure S3. MMP15 does not correlate with maternal BMI in early pregnancy.** MMP15 expression (**A**) and protein levels (pro-MMP15, active (act)-MMP15 and total-MMP15: pro+act-MMP15, **B**-**D**) were determined in first trimester placental tissue from lean (GW 5-11, n = 24) and obese (GW 5-10, n = 18) women by RT-qPCR and Western blotting, respectively. Results were normalized to HLA-G expression (–ΔCt) and protein levels. Pearson’s or Spearman’s correlations were used to determine associations between MMP15 and maternal BMI.


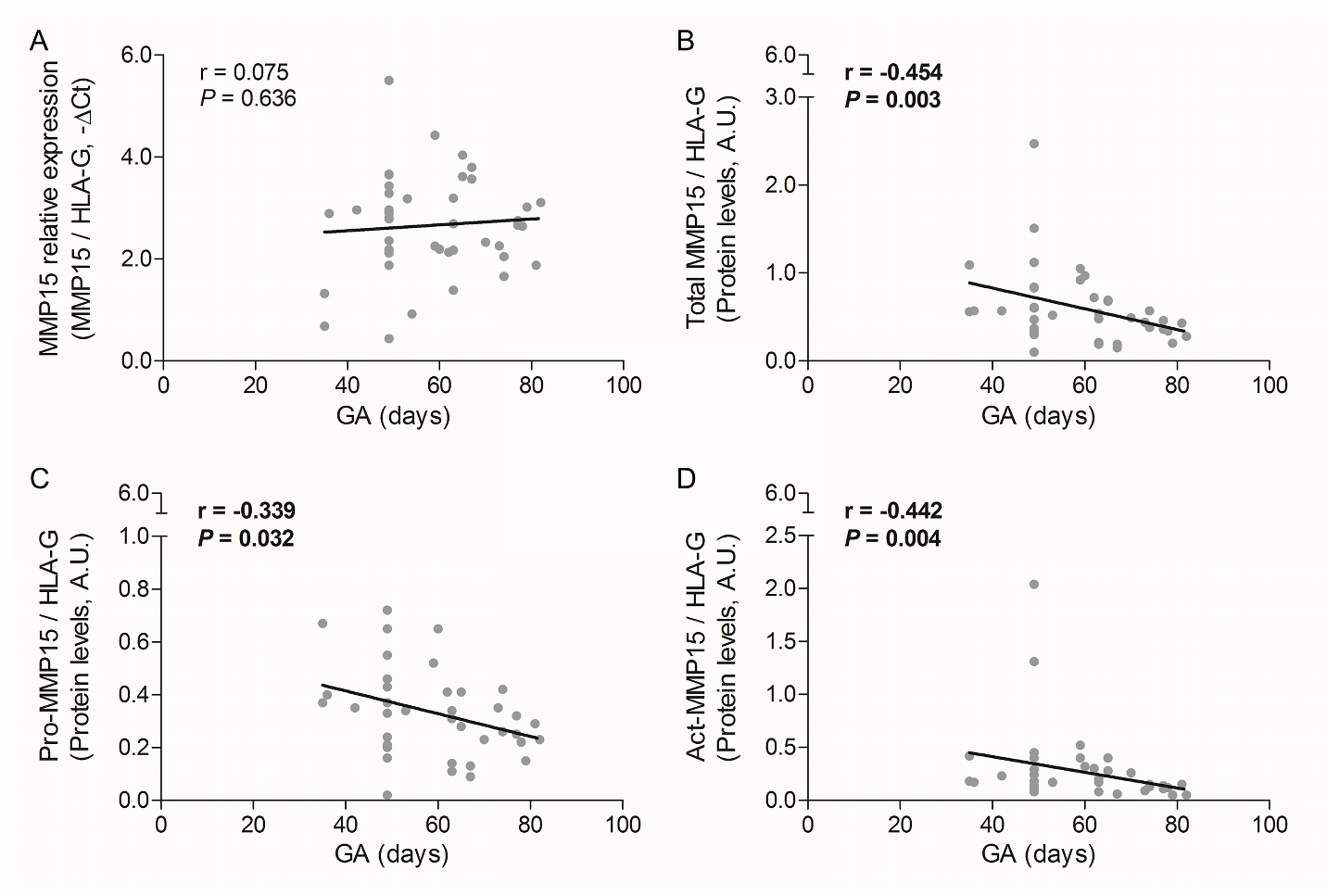


**Supplementary Figure S4. MMP15 protein levels negatively correlate with maternal gestational age (GA) in early pregnancy.** MMP15 expression (**A**) and protein levels (pro-MMP15, active (act)-MMP15 and total-MMP15: pro+act-MMP15, **B**-**D**) were determined in first trimester placental tissue (GW 5-11, n = 42) by RT-qPCR and Western blotting, respectively. Results were normalized to HLA-G expression (–ΔCt) and protein levels. Pearson’s or Spearman’s correlation were used to determine associations between MMP15 and GA.
