# Supplementary material for: Matrix metalloproteinase 15 plays a pivotal role in human first trimester cytotrophoblast invasion and is not altered by maternal obesity
Source: FASEB J. 2020 Jul 2;34(8):10720–30. doi: 10.1096/fj.202000773R (PMC7496590; doi:10.1096/fj.202000773R)
Supplement: Supplementary file 2 — Table S1 [file FSB2-34-10720-s002.docx]

**Supplementary Table S1**. Primary and secondary antibodies used in the study.

| **Antibody** | **Company** | **Cat. #** | **Dilution** | **Species** | **Application** |
| --- | --- | --- | --- | --- | --- |
| Caspase-cleaved cytokeratin 18 | Roche | 14533800 | 1:25 | Mouse | IF |
| Cytokeratin 7 | Santa Cruz | C2206 | 1:100 | Mouse | IF |
| Cytokeratin 7 | Ventana | 790-4462 | 1:75 | Rabbit | IF |
| HLA-G | Exbio | 11449-C100 | 1:100 | Mouse | IF |
| HLA-G | BD Biosciences | 557577 | 1:1000 | Mouse | WB |
| Ki67 | Thermo Scientific | RM-9106-S0 | 1:100 | Rabbit | IF |
| MMP15 | Millipore | MAB3320 | 1:500 / 1:1250 | Mouse | WB / IF |
| Alexa Fluor anti-mouse 488 | Thermo Scientific | 11001 | 1:300 | Goat | IF |
| Alexa Fluor anti-mouse 568 | Thermo Scientific | 11004 | 1:300 | Goat | IF |
| Alexa Fluor anti-rabbit 488 | Thermo Scientific | 11008 | 1:300 | Goat | IF |
| Alexa Fluor anti-rabbit 568 | Thermo Scientific | 11011 | 1:300 | Goat | IF |
| Anti-mouse HRP-conjugated | BioRad-Laboratories | 1706516 | 1:1000 / 1:2000 | Goat | WB |

IF: immunofluorescence; WB: Western blotting.
